# Lichen Planus of the Eyelids: Case Report of a Rare Presentation of a Common Dermatosis

**DOI:** 10.7759/cureus.57299

**Published:** 2024-03-30

**Authors:** Nouf M AlEid, Omotoyosi N Ilesanmi, Mona A Alshehri, Ahmed A Alhumidi, Hind AlShihry

**Affiliations:** 1 Dermatology and Dermatologic Surgery, Prince Sultan Military Medical City, Riyadh, SAU; 2 Dermatology, University of Ilorin Teaching Hospital, Ilorin, NGA; 3 College of Medicine, King Saud Bin Abdulaziz University for Health Sciences, Riyadh, SAU; 4 Pathology, King Saud University, Riyadh, SAU

**Keywords:** eruption, pruritic, dermatosis, eyelid, lichen planus

## Abstract

Lichen planus (LP) is a common T-cell-mediated autoimmune skin disease, and its exact etiology is unknown. Typically, it affects the trunk, flexural surfaces, and the mucosa.We report a rare finding of LP involving both eyelids in a 67-year-old female.

A 67-year-old Saudi female with a medical history of diabetes mellitus, hypothyroidism and rheumatoid arthritis presented with a three-month history of pruritic skin eruptions in both eyelids. She had no associated musculoskeletal symptoms or fatigue and no medical or family history of atopy. The patient had violaceous, thin, scaly plaques confined to both eyelids. Oral mucosa, genitalia, scalp, and nails were not affected. Histopathology from the right lower eyelid confirmed the diagnosis of LP. Hepatitis C virus serology was negative. Patient was examined by ophthalmology to rule out conjunctival involvement of LP. She had dry eyes only. She was initially managed by topical tacrolimus 0.1% ointment and didn't tolerate it due to severe reaction. She tolerated mometasone propionate 0.1% cream, which relieved the itch and partially improved the lesions.

Although rare, LP of the eyelids must be considered among differential diagnoses of eyelid dermatitis. It can be confined, or it may concomitantly involve other parts of the body. LP of the eyelid may also extend to the conjunctiva, so it's important to screen patients by ophthalmology to rule out possible ocular involvement. This is the first case report of a Saudi patient with LP confined to the eyelid. The management of LP involving the eyelids is challenging. Treatment options include topical steroids, tacrolimus ointment, phototherapy and oral retinoids (etretinate).

## Introduction

Lichen planus (LP) is a common inflammatory skin disease. It is a T-cell-mediated autoimmune reaction and its exact etiology is unknown. It is characterized by violaceous, flat-topped pruritic papules, with a network of white fine lines on its surface known as ‘Wickham striae’. The lesion presents with focal hypergranulosis, due to thickening of the granular layer on histology [1]. Lichen planus usually affects the trunk, flexural surfaces and oral or genital mucosa [2]. Eyelid is rarely affected, with only a few reported cases [3]. We report an isolated case of lichen planus involving both eyelids. This article was previously presented as a poster abstract in 2019 at the American Academy of Dermatology Annual meeting.

## Case presentation

This is a 67-year-old lady with a background history of diabetes mellitus, hypertension, and hypothyroidism. Her chronic diseases are well controlled on medications. She came to the outpatient dermatology clinic with a three-month history of pruritic skin eruptions confined to the upper and lower eyelids bilaterally. The lesions increased gradually over the three-month period before she sought care for them in our clinic. There were no associated systemic manifestations such as a history of fever, malaise, weight loss, muscle weakness, arthralgia or fatigue. The lesions did not exacerbate or improve by sun exposure. There were no other skin lesions. She was not in contact with other people who had a similar condition to hers. There was no travel history, and she did not use any topical products (medical or herbal) two weeks prior to developing this rash.

A systematic review revealed no cardiovascular, respiratory, neurological, gastrointestinal or genitourinary symptoms. Past surgical history was unremarkable. Her current medications are the following: gliclazide 60mg orally (PO) twice daily (BD), empagliflozin 25mg PO once daily (OD), amlodipine 5mg PO OD, aspirin 81mg PO OD, atorvastatin 20mg PO OD, vitamin b complex supplements and levothyroxine 100mcg PO OD. All her oral medications started over five years ago. There was no personal or family history of atopy. She lives in Riyadh city with her family. She doesn’t smoke, or consume alcohol or other recreational drugs. She did not seek dermatology consultation for this eruption before. 

**Figure 1:**
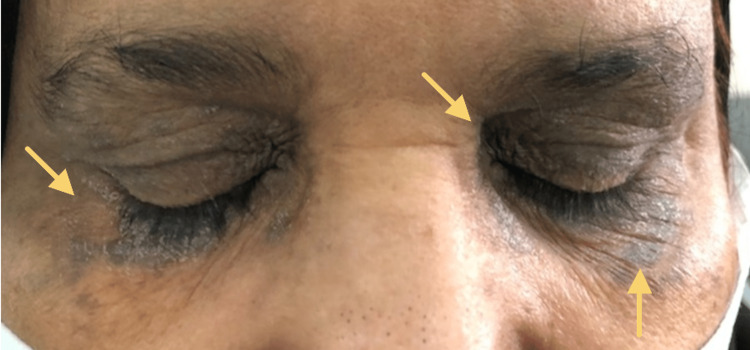
Multiple well-defined violaceous, thin papules and plaques with a white scale over both eyelids bilaterally.

On examination, the patient was vitally stable, alert, conscious and not in pain. Skin examination revealed multiple well-defined violaceous, thin, and scaly plaques over both upper and lower eyelids bilaterally (Figure 1). The mucosal membranes exam revealed no involvement of oral, ocular, genital, or anal mucosa. The scalp and nails were not affected. No other lesions were found on the skin. There were no target or targetoid lesions, and Nikolsky sign was negative. The lymph node exam was unremarkable.

**Figure 2:**
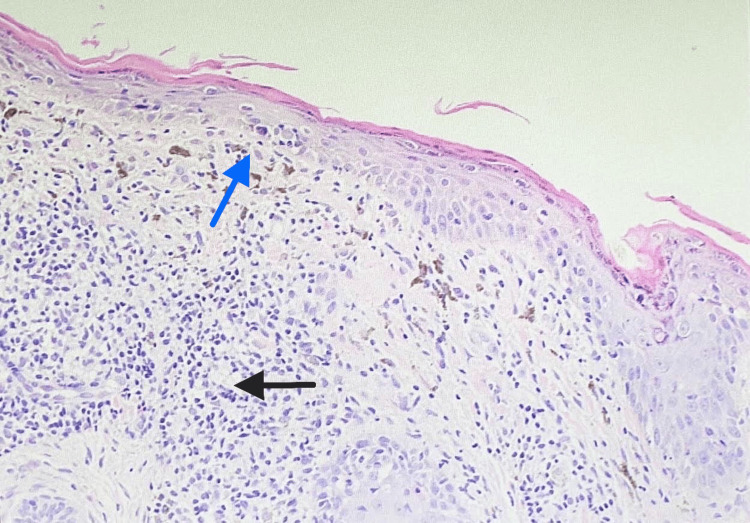
Scanning power view of biopsied skin shows a lichenoid reaction pattern characterized by the combination of epidermal atrophy, degeneration of the basal layer of the epidermis (blue arrow), and interface lymphocytic infiltrate involving the superficial dermis (black arrow).

**Figure 3:**
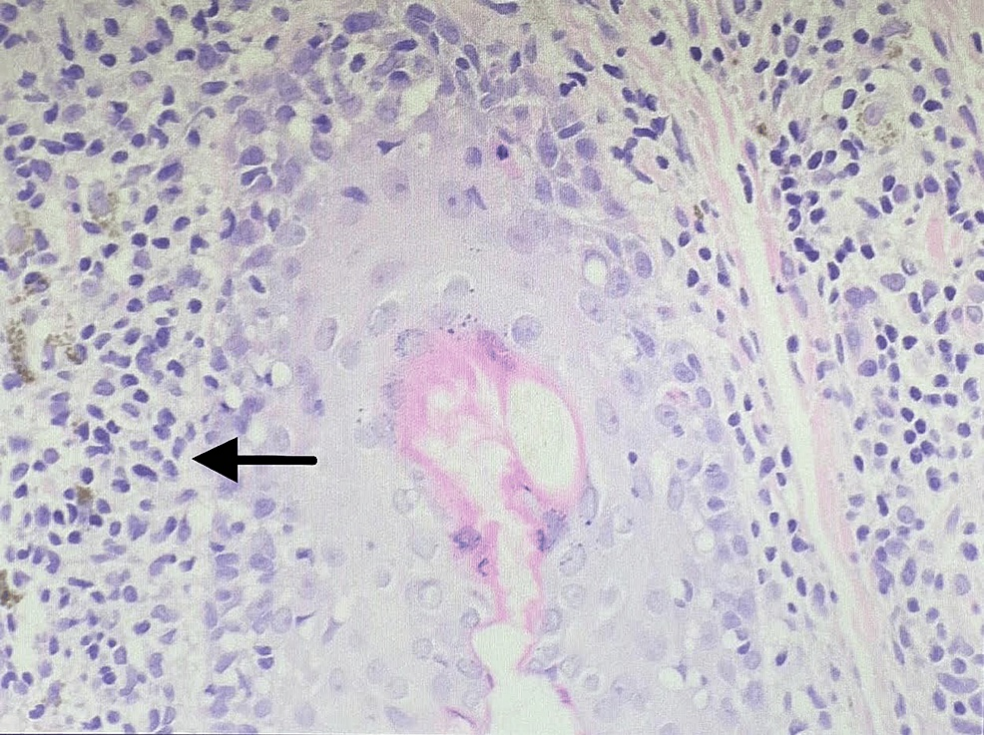
Scanning power view of the same skin biopsy shows a lichenoid reaction pattern; interface lymphocytic infiltrate surrounding hair follicles.

Next step in her assessment was obtaining a skin biopsy for microscopic examination using H&E stain. A 3 mm skin punch biopsy of the right lower eyelid was taken under local anesthesia. Biopsy results showed the typical changes of lichen planus (Figures 2, 3). Her laboratory investigations including complete blood count, renal and liver function tests were within normal ranges. Her hemoglobin A1c was 7.62% which is within target of diabetic patients' control. Serology testing for hepatitis C virus was negative. Since the lichen planus lesions are affecting the skin of the eyes, the patient needed to be assessed by an ophthalmologist. Their assessment ruled out conjunctival involvement, and diagnosis of dry eyes was made.

Treatment options for eyelid lichen planus are limited due to the thin skin in the area. We started our patient on topical 0.1% tacrolimus ointment twice daily, however she developed severe irritation in the affected skin. We changed to topical 0.1% mometasone propionate cream twice daily, which relieved pruritus. She was reassessed during her follow-up visits. The lesions improved partially but did not completely resolve.

## Discussion

While lichen planus of the eyelids is rare, it should be considered in the differential diagnosis of eyelid dermatitis. Differential diagnosis includes contact dermatitis, atopic eczema, psoriasis, seborrheic dermatitis, acne rosacea, chronic cutaneous lupus erythematosus, dermatomyositis, and microbial infections (viral, bacterial, and fungal). Allergic contact dermatitis has been identified as the most common differential diagnosis [4].

Lichen planus of the eyelids can be found in isolation, or it may concomitantly involve other parts of the body. Lichen planus may also affect the conjunctiva in addition to the eyelids, leading to cicatricial conjunctivitis [5]. Therefore, patients with lichen planus of eyelids require an ophthalmologic examination to rule out ocular involvement.

This is the first case report of a Saudi patient with lichen planus confined to the eyelid. To the best of our knowledge and exploration through PubMed, this is the 20th reported case of LP of the eyelid in English literature [3]. Both Michelson in 1937 and Sharma in 2001 described five separate cases [6,7]. As per literature search, the most recent case was reported by Huang in 2016 [8] where he reported a case of LP involving the eyelids, chest, and lower extremities. This case differs from the latter in the affected site. In this case, lesions exclusively affected the eyelids, posing a clinical diagnostic challenge. In presenting this rare case we hope to share our findings and encourage widening the scope of differential diagnoses for eyelid dermatoses. The management of lichen planus involving the eyelids is challenging. Options include the use of topical steroids, tacrolimus ointment, phototherapy, and the use of oral retinoids (etretinate or acitretin).

## Conclusions

Although rare, LP of the eyelids must be considered among differential diagnoses of eyelid dermatitis. It can be confined, or it may concomitantly involve other parts of the body. LP of the eyelid may also extend to the conjunctiva, so it's important to screen patients by ophthalmology to rule out possible ocular involvement. This is the first case report of a Saudi patient with LP confined to the eyelid. The management of LP involving the eyelids is challenging. Treatment options include topical steroids, topical calcineurin inhibitors (tacrolimus ointment), phototherapy, and oral retinoids (acitretin or etretinate).
